# The Impact of Training in Multimodal Communication Skills on Psychotropic Medication Use in Dementia Care

**DOI:** 10.7759/cureus.63413

**Published:** 2024-06-28

**Authors:** Masaki Kobayashi, Saki Une, Hisao Hara, Miwako Honda

**Affiliations:** 1 Internal Medicine, Unity Hospital, Rochester Regional Health, Rochester, USA; 2 General Internal Medicine, Division of Geriatric Research, National Hospital Organization Tokyo Medical Center, Tokyo, JPN; 3 Internal Medicine, Koriyama Medical Care Hospital, Koriyama, JPN

**Keywords:** elderly patients, psychotropic drugs, long term care, dementia care, interpersonal and communication skills

## Abstract

Aim

This study aimed to assess the trends in psychotropic drug prescriptions among elderly residents with dementia following the continuous implementation of multimodal comprehensive care communication skills training for staff in a long-term care facility.

Methods

This retrospective single-center cross-sectional study utilized the database of an urban public hospital that included a long-term care facility. The data were collected from 2016 to 2020. All 130 staff members at the hospital (52 nurses, 48 professional caregivers, seven rehabilitation staff members, three physicians, and three pharmacists) initiated multimodal comprehensive care communication skills basic training from October 2014 to December 2015, which was followed by continuous monthly training until the end of 2020. Antipsychotic prescription rates for residents aged over 65 years with dementia were measured throughout the study period.

Results

A total of 506 eligible residents were identified, the median age was 86.0 years (IQR: 81.0-90.0), and 283 (55.9%) residents were females. The prescription rates for psychotropic drugs among residents with dementia decreased significantly (43.5% in 2016, 27.0% in 2020; p=0.01). Notably, the percentage of patients prescribed anxiolytics decreased significantly (from 4.7% to 0.0%), while the percentage of patients receiving antipsychotic drugs, hypnotics, antidepressants, or antiepileptic drugs remained unchanged over time. The prescription rates for antidementia drugs significantly decreased from 15.3% to 4.0%.

Conclusion

The prescription rates of psychotropic drugs were significantly reduced following multimodal comprehensive care communication skills training for staff at a long-term care facility. The improvement in communication skills among staff at long-term care facilities has a tangible impact on reducing drug use among elderly residents with dementia.

## Introduction

Dementia is a syndrome that involves deterioration in cognitive function beyond what might be expected from the usual consequences of biological aging [1]. Currently, more than 55 million people live with dementia worldwide, and nearly 10 million new cases are diagnosed every year [1]. Behavioral and psychological symptoms of dementia (BPSDs) are crucial features in the clinical course of dementia in older adults [2]. BPSDs include agitation, depression, apathy, psychosis, aggression, sleep problems, wandering, and a variety of inappropriate behaviors [3]. One or more of these symptoms will affect nearly all people with dementia over the course of their illness [3]. BPSDs are also associated with earlier institutionalization, and ultimately, most residents in long-term care facilities have dementia [4].

Although nonpharmacologic approaches should be used as the first-line treatment for managing BPSDs [3], psychotropic medications (i.e., antipsychotic drugs, sedatives/hypnotics, antidepressants, and benzodiazepines) are commonly administered to older adults with dementia in long-term care [5]. However, older adults are highly vulnerable to the adverse effects of psychotropic medications [5]. Antipsychotic drugs are often prescribed with the aim of controlling BPSDs in older adults with dementia; however, the prescription of antipsychotic medication may be an ineffective and potentially dangerous strategy because of side effects, including increased risk for mortality and cerebrovascular events [6]. Antipsychotic drugs, antidepressants, and benzodiazepines are consistently associated with a greater risk of falls in older adults [7]. The incidence and prevalence of antiepileptic drug (AED) use among people with dementia are high [8]. While AEDs are used not only for seizure control but also for neuropathic pain, migraine prophylaxis, and control of BPSDs, AED users with dementia have been found to have an increased relative risk of death and cerebrovascular events compared with nonusers with dementia [8]. Administering psychotropic drugs is often viewed as a reliable solution to control behaviors when staff are faced with time restraints, have minimal knowledge of behavioral approaches, and perceive that psychotropic drugs are efficacious and low risk [9].

A previous systematic review and meta-analysis indicated that psychosocial training and support for nursing home staff significantly decreased the prescription rate of antipsychotic drugs. However, no study has shown a significant reduction in the prescription of psychotropic drugs, including sedatives/hypnotics, antidepressants, and benzodiazepines [10]. A program with a person-centered care approach in the United Kingdom showed a significant reduction in the proportion of residents receiving antipsychotics in the intervention group (23.0%) compared to the control group (42.1%) after 12 months [11]. On the other hand, the implementation of a person-centered care approach did not reduce antipsychotic prescriptions in German nursing homes [12]. A study in Germany reported that a person-centered care approach was not implemented to the desired extent due to differences in the health care systems in the United Kingdom and Germany and contextual factors such as staff and time constraints [12]. To our knowledge, there is a lack of research on effective interventions to significantly reduce psychotropic drug prescriptions in older adults with dementia in long-term care.

Previous studies have suggested searching for the underlying causes of BPSDs and applying nonpharmacological interventions, including staff training and environmental changes, prior to prescribing psychotropic drugs [13]. Moreover, interventions that aim to improve prescription should involve a broad approach that targets the skills of care staff, strong communication, collaboration, and equitable decision-making [14]. In particular, staff communication during dementia care and the relationship between staff and residents directly affect residents’ challenging behaviors [15]. A previous study evaluated a staff communication training program that included in-person training sessions with videos, vignettes, and role-playing focused on fulfilling resident communication needs, identifying and reducing elderspeak, and practicing effective dementia communication practices [16]. Education training significantly decreased antipsychotic medication use from 20.7% to 15.8% [16]. However, this study focused on antipsychotic drugs and may have included residents without dementia [16].

Several studies have shown that training health care workers in multimodal communication skills has the potential to reduce BPSD and improve the overall well-being of individuals with dementia [17]. One notable care methodology called Humanitude, developed by Gineste and Marescotti in 1979 [18,19], offers a multimodal communication technique based on a humanist philosophy that emphasizes respect for individual liberty, autonomy, and dignity [18]. Humanitude adopts a relationship-centered and compassionate care approach, focusing on the following four pillars: gaze, talk, touch, and assistance with standing up and providing care in one sequence divided into five steps [18]. This methodology has demonstrated its effectiveness in reducing agitation and psychological symptoms among individuals with dementia. However, to date, no study has specifically evaluated the effect of this methodology on medication use for people with dementia.

The purpose of this study was to analyze the effect of a multimodal comprehensive care methodology training program on the use of psychotropic drugs in a long-term care facility. The training program was provided to all staff members in a long-term care institution. By investigating the impact of the training program on medication use, we aimed to gain insights into the potential benefits of the methodology in reducing the reliance on psychotropic drugs and improving the overall well-being of individuals with dementia.

## Materials and methods

Study design and setting

A retrospective single-center cross-sectional design was used to collect data from January 2016 to December 2020 from the database of Koriyama Medical Care Hospital. Located in Koriyama, Fukushima Prefecture, Japan, this hospital serves as a 120-bed long-term care facility. Throughout the study period, the hospital employed internal medicine physicians and part-time psychiatric physicians but lacked geriatricians.

From October 2014 to December 2015, all staff members (including three physicians, 52 nurses, 48 professional caregivers, seven rehabilitation staff, three pharmacists, and 15 clerks) underwent the basic training program of the multimodal comprehensive care methodology. In this study, a multimodal comprehensive care methodology training program was developed for all staff at a long-term care institution to teach the multimodal communication skills used in long-term care. The program includes lectures on the philosophy of the methodology and the basics of communication skills, demonstrations, video learning, role-playing workshops, and bedside training. This training is designed to equip staff with the necessary skills to care for residents with dementia in a long-term care facility. This training was facilitated by three certified instructors, consisting of one nurse and two professional caregivers, all of whom completed a 10-week training program on teaching Humanitude. Following the initial training, hospital staff received monthly continuous training in the methodology, continuing through the end of 2020.

Data collection and participants

The data were collected using the electronic medical records system of Koriyama Medical Care Hospital. Information on age, sex, medical history, care need levels under the public long-term care insurance system in Japan, and medication use during hospitalization was retrieved from electronic medical records. The study included residents aged 65 years or older who were admitted to the hospital between January 2016 and December 2020 and who had a diagnosis of dementia. Residents who were admitted multiple times during the study period were treated as separate residents. The need for individual informed consent was formally waived by the institutional medical ethics committee because only data from medical records were used and the patients were not contacted directly. However, we displayed an opt-out statement on the webpage of the hospital to inform patients about the study and to provide the opportunity for patients to decline the use of their data. Three researchers (MH, MK, SU) reviewed and analyzed the information on age, sex, care need levels, medication use during hospitalization, and the Charlson Comorbidity Index (CCI) [20]. Regarding medication data, we reviewed information on regularly scheduled and as-needed medications prescribed for residents during their admission.

Psychotropic drugs included five categories based on the anatomical therapeutic chemical classification system: antipsychotics, antidepressants, hypnotics, anxiolytics, and antiepileptic drugs [21]. Antidementia drugs consisted of three acetylcholinesterase inhibitors (donepezil, galantamine, and rivastigmine) and one N-methyl-D-aspartate receptor antagonist (memantine). In this study, the collected data on drug use included first-time, chronic, and as-needed prescriptions. The study protocol was approved at the National Hospital Organization Tokyo Medical Centre and the Koriyama Medical Care Hospital.

Outcome measures

The primary outcome of this study was the annual change in prescription rates of psychotropic drugs for residents admitted from 2016 to 2020. The secondary outcome was the annual change in prescription rates of specific categories of psychotropic drugs and antidementia drugs for elderly residents admitted from 2016 to 2020.

Statistical analysis

The characteristic data of residents with dementia were analyzed using descriptive statistics. Nonnormally distributed quantitative variables are presented as medians and interquartile ranges (IQRs). The normality of the data was verified using the Shapiro-Wilk test. Analytical statistics were used to assess the primary and secondary outcomes. Changes in the prescription rates of each medication for residents admitted from 2016 to 2020 were tested using the chi-square test. P<0.05 indicated statistical significance. The prescription rates were compared between 2016 and 2020 using the chi-square test. These analyses were performed using R statistical software version 4.0.2 (Vienna, Austria: R Foundation for Statistical Computing).

## Results

A total of 506 residents were enrolled in this study, and the demographic characteristics of the residents are detailed in Table 1. Among these residents, 283 (55.9%) were women. The median age was 86.0 years (IQR: 81.0-90.0). Specifically, 157 (31.0%) residents were aged 75-84 years, while 307 (60.7%) were over 85 years of age. The median Charlson Comorbidity Index (CCI) was 2.0 (IQR: 1.0-4.0), indicating prevalent comorbidities among the participants.

**Table 1:** Characteristics of the residents with dementia.

Characteristics	Observed data, n=506
Women, n (%)	283 (55.9)
Age, n (%)	
65-74 years	42 (8.3)
75-84 years	157 (31.0)
≥85 years	307 (60.7)
Average, median (IQR)	86.0 (81.0-90.0)
Care need level, n (%)	
Level 1	17 (3.4)
Level 2	21 (4.2)
Level 3	56 (11.1)
Level 4	187 (37.0)
Level 5	203 (40.1)
Charlson Comorbidity Index, median (IQR)	2 (1-4)

In Japan, the public long-term care insurance system categorizes frail older adults into five levels of care need (levels 1-5), with higher numbers indicating more severe needs. This classification is determined using a nationally standardized and validated algorithm that evaluates both physical and mental care needs [22]. Research has shown that older adults categorized at higher care need levels are more likely to be institutionalized than those classified at level 1 or 2 [23]. In our study, 203 residents (40.1%) were categorized as having a level of 5, while 187 residents (37.0%) had a level of 4, underscoring the elevated care requirements among the enrolled residents.

Trends in psychotropic drug use

**Figure 1:**
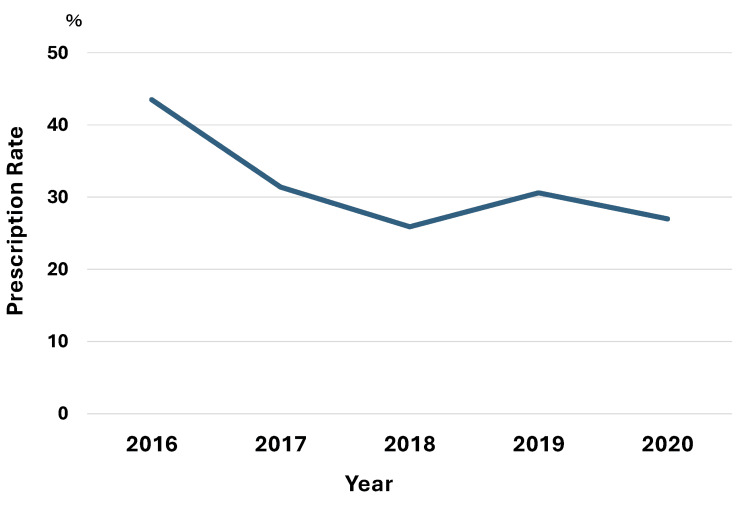
The annual change in prescription rates of psychotropic drugs for elderly residents admitted from 2016 to 2020.

Table 2 shows the trends in prescription rates of psychotropic and antidementia drugs among residents. The prescription rates indicated the proportions of residents who were prescribed psychotropic and antidementia drugs each year. In this study, some residents were prescribed more than two psychotropic drugs simultaneously. The prescription rates for psychotropic drugs decreased significantly from 37 out of 85 (43.5%) in 2016 to 34 out of 126 (27.0%) in 2020 (p=0.01) as illustrated in Figure 1.

**Table 2:** Trends in psychotropic and antidementia drug prescription rates for residents (2016-2020). Statistical analysis was performed using the chi-squared test. P≤0.05 was considered statistically significant.

Variables	2016 (n=85)	2017 (n=70)	2018 (n=81)	2019 (n=144)	2020 (n=126)	Chi-square	p-Value
Psychotropic drugs, n (%)	37 (43.5)	22 (31.4)	21 (25.9)	44 (30.6)	34 (27.0)	6.22	0.01
Antipsychotics, n (%)	6 (7.1)	8 (11.4)	8 (9.9)	12 (8.3)	12 (9.5)	0.40	0.53
Hypnotics, n (%)	17 (20.0)	9 (12.9)	10 (12.3)	17 (13.9)	15 (15.9)	2.59	0.11
Antidepressants, n (%)	7 (8.2)	2 (2.9)	2 (2.5)	3 (2.1)	5 (4.0)	1.72	0.19
Anxiolytics, n (%)	4 (4.7)	2 (2.9)	1 (1.2)	3 (2.1)	0 (0.0)	6.04	0.01
Antiepileptic drugs, n (%)	12 (14.1)	7 (10)	10 (12.3)	22 (15.2)	12 (9.5)	1.06	0.3
Antidementia drugs, n (%)	13 (15.3)	17 (24.3)	7 (8.6)	8 (5.6)	5 (4.0)	8.34	0.003

The breakdown of psychotropic drugs reveals that the prescription rates for anxiolytics decreased significantly from 4 out of 85 (4.7%) in 2016 to 0 out of 126 (0.0%) in 2020 (p=0.01). The prescription rates for antipsychotics slightly increased from 6 out of 85 (7.1%) in 2016 to 12 out of 126 (9.5%) in 2020; however, this difference was not statistically significant (p=0.53).

The prescription rates for hypnotics, antidepressants, and antiepileptics decreased from 17 out of 85 (20.0%) to 15 out of 126 (15.9%), from 7 out of 85 (8.2%) to 5 out of 126 (4.0%), and from 12 out of 85 (14.1%) to 12 out of 126 (9.5%), respectively, between 2016 and 2020. However, these decreases were not statistically significant. Moreover, the prescription rates for antidementia drugs significantly decreased from 13 out of 85 (15.3%) in 2016 to 5 out of 126 (4.0%) in 2020 (p=0.003).

## Discussion

This retrospective cross-sectional study offers significant insights into the impact of comprehensive multimodal care communication training for staff in long-term care facilities on psychotropic drug prescription rates for elderly residents with dementia.

Our study demonstrated a notable decrease in the prescription rates of psychotropic drugs among elderly residents with dementia following comprehensive multimodal communication training implemented for all staff members. At the outset of our study in 2016, the prescription rate of psychotropic drugs was 43.5%, aligning with the rates reported in a prior study focused on residents with dementia in Japanese long-term care facilities [24]. A previous study on standard geriatric care reported no significant change in drug utilization over a two-month period, with rates remaining stable between 44.3% and 42.8% [24]. In contrast, our study revealed a notable trend over the study duration, with the prescription rate of psychotropic drugs decreasing significantly from the initial 43.5% to 27.0% in the four-year period. It is essential to note that our multimodal care communication skills training initiative primarily aimed to enhance communication strategies, reduce agitation and resistance to care among residents with dementia, and foster improvements in their overall well-being, participation, and mobility rather than specifically targeting psychotropic drug use reduction [17,18]. The reason for evaluating psychotropic drug use after communication training is related to the preventive nature of this training in reducing the challenging behaviors of people with dementia that frequently lead to psychotropic drug use. Previous studies have indicated that effective communication strategies can significantly diminish episodes of agitation and aggression in dementia patients [17]. By equipping staff with improved communication skills, a prior study showed a reduction in such behaviors, potentially decreasing the subsequent need for psychotropic medications to manage these symptoms [17].

Crucially, our study stands apart from prior research by extending the intervention to encompass all staff members within the long-term care facility rather than focusing solely on specific professional groups. Unlike earlier studies that targeted training towards select staff categories such as physicians, nurses, or pharmacists [10,13], our approach involved training the entire staff responsible for resident care. This collaborative engagement among all stakeholders as equal partners can effectively diminish inappropriate antipsychotic prescriptions [9]. Previous studies have indicated that managerial support, consistent staff routines, shared understanding among team members, and interprofessional collaboration are pivotal factors that promote culture change and streamline the deprescribing of psychotropic drugs for dementia patients within nursing home environments [14,25]. By engaging all staff members in our communication training initiative, our objective was to foster a unified understanding and collaborative synergy. This approach aimed to cultivate a holistic and integrated care strategy for residents with dementia.

This was a retrospective single-center study, not a cluster randomized controlled trial targeting nursing homes. However, a previous cohort study evaluating the prescription of psychotropic drugs in 1201 residents with dementia admitted to 343 long-term care facilities in Japan showed that the utilization of psychotropic drugs did not change during the study period [24]. Our study suggested a potential clinical impact of communication training on the use of psychotropic drugs among dementia patients. Further randomized controlled trials spanning multiple facilities are essential to corroborate the link between communication training and psychotropic drug usage in this demographic.

The discussion also highlights the prevalence and prescription rates of specific types of psychotropic drugs. Our findings revealed consistent prescription rates for antipsychotic drugs, aligning with prior research on person-centered care approaches [12]. Nevertheless, the prevalence of antipsychotic drug use in our study was notably lower than that in studies on person-centered care from other countries [11,12]. These disparities may stem from differing care methodologies, such as the relationship-centered care advocated by the Humanitude approach in our research, coupled with variations in staffing and time constraints. Person-centered care emphasizes recognizing the unique individuality of each person in every facet of care, tailoring both care practices and environments to suit individual needs, and understanding behaviors from the perspective of the person with dementia. In contrast, Humanitude prioritizes relationship-centered care [17,18]. The primary objective of the Humanitude approach is to cultivate strong bonds between caregivers and care recipients, fostering a shared sense of meaningful engagement throughout their interactions [17,18]. Notably, staff often find greater satisfaction in their caregiving roles when they perceive their caregiving techniques as impactful. Notably, due to challenges such as limited staffing and time constraints, relationship-centered care as promoted by Humanitude is sometimes viewed as more feasible for staff compared to the intensive demands of person-centered care, a sentiment echoed in various studies from Japan and beyond [17].

Furthermore, our study showed varying patterns of prescription rates for hypnotics, anxiolytics, antidepressants, and antiepileptic drugs (AEDs). In our study, hypnotics were found to be frequently prescribed among psychotropic drugs and their prescription rates did not significantly change from 2016 to 2020; however, the prevalence of hypnotics decreased (20% in 2016 to 15.9% in 2020). Fifteen percent of residents in this study were prescribed hypnotics in 2020, which was slightly lower than that reported in another study in Japan (22.6-25.1%) [24]. However, given the prevalence of hypnotics reported in studies in Germany and Austria (9.9-13.3%), our data on hypnotics in long-term care may suggest the importance of the appropriate use of hypnotics in long-term care [26]. The prescription rates for anxiolytics significantly decreased over time in our study. The prevalence of anxiolytic use in our study was lower than that in a previous study in Japan and other countries [24,26]. One possible reason for the 4.7% prevalence of anxiolytic use in 2016 could be the proactive efforts of the hospital pharmacist to promote appropriate anxiolytic usage following the 2014 communication training. Although the prescription rates of antidepressants remained statistically consistent throughout our study, declining from 8.2% in 2016 to 4.0% in 2020, this trend aligns with findings from a meta-analysis of five studies that similarly found no significant impact of psychosocial interventions on antidepressant use [10]. Notably, our study's antidepressant prescription rate was substantially lower than that in other international studies, where approximately one-third of nursing home residents with dementia were prescribed antidepressants [13,27]. Although our study revealed a decrease in the use of antiepileptic drugs (AEDs) from 14.1% in 2016 to 9.5% in 2020, the initial prevalence of AEDs in 2016 was markedly higher than that in another Japanese study, which ranged from 7.1% to 7.8% [24]. Given recent U.S. research indicating a rising trend in AED prescriptions among dementia-afflicted nursing home residents, our data underscore the critical need for judicious AED use in long-term care and highlight the potential benefits of comprehensive communication training for dementia care staff [28].

Finally, our study revealed a decrease in the prescription rates of antidementia drugs during the study period. This finding contrasts with studies conducted in Denmark and the United Kingdom that reported increased prescription rates [29,30]. We speculate that the differences may be attributed to the management of behavioral and psychological symptoms of dementia (BPSDs) in our study, as antidementia drugs are used not only to improve cognitive function but also to address BPSDs. Furthermore, most of the patients in our study had moderate to severe dementia, given that approximately 80% of them had a care need level of 4 or 5. The severity of dementia and individual tolerability may influence the cessation of antidementia drug use in residents with severe dementia.

Several limitations should be discussed. First, the retrospective cross-sectional design means that potential confounders, such as patient characteristics or staff training levels, might influence the observed relationship between communication training and psychotropic drug prescription rates. Although we did not pinpoint these factors, future research should explore them further. Second, being a single-center study with a limited sample size could account for some nonsignificant findings. Third, we were unable to compare the data between chronic and as-needed use of antipsychotics because we collected data on first-time, chronic, and as-needed prescriptions without capturing the reasons for prescribing psychotropic drugs to the residents. Finally, while many psychotropic drugs might be unsuitable for older adults, our study could not assess the appropriateness of prescribed medications due to data constraints.

## Conclusions

The present study contributes to the growing body of evidence linking multimodal comprehensive care communication training for staff in long-term care facilities to reduced psychotropic drug use among elderly residents with dementia. Despite the limitations inherent in our retrospective, single-center design, the findings highlight the potential benefits of this training approach, emphasizing its role in fostering relationship-centered care. To validate and expand upon our findings, future research, particularly randomized controlled trials involving multiple facilities, is essential.
